# Variability in emotional/behavioral problems in boys with oppositional defiant disorder or conduct disorder: the role of arousal

**DOI:** 10.1007/s00787-015-0790-5

**Published:** 2015-11-25

**Authors:** Jantiene Schoorl, Sophie Van Rijn, Minet De Wied, Stephanie H. M. Van Goozen, Hanna Swaab

**Affiliations:** 1 Department of Clinical Child and Adolescent Studies, Leiden University, Wassenaarseweg 52, PO Box 9555, 2300 RB Leiden, The Netherlands; 2 Leiden Institute for Brain and Cognition, Leiden University, Leiden, The Netherlands; 3 Department of Adolescent Development, Utrecht University, Heidelberglaan 1, PO Box 80140, 3508 TC Utrecht, The Netherlands; 4 School of Psychology, Cardiff University, PO Box 901, Cardiff, CF103YG Wales UK

**Keywords:** Heart rate variability, Arousal, Reactive aggression, Proactive aggression, Psychopathology, Children

## Abstract

It is often reported that children with oppositional defiant disorder (ODD) or conduct disorder (CD) are under-aroused. However, the evidence is mixed, with some children with ODD/CD displaying high arousal. This has led to the hypothesis that different profiles of arousal dysfunction may exist within children with ODD/CD. This knowledge could explain variability within children with ODD/CD, both in terms of specific types of aggression as well as comorbid symptoms (e.g., other emotional/behavioral problems). We measured heart rate variability (HRV), heart rate (HR) and skin conductance level (SCL) during rest and stress, and obtained parent and teacher reports of aggression, anxiety, attention problems and autism traits in a sample of 66 ODD/CD and 36 non-clinical boys (aged 8–12 years). The ODD/CD group scored significantly higher on aggression, anxiety, attention problems and autism traits than the controls; boys with ODD/CD also had higher resting HRs than controls, but HR stress, HRV and SCL did not differ. Hierarchical regressions showed different physiological profiles in subgroups of boys with ODD/CD based on their type of aggression; a pattern of high baseline HR and SCL, but low stress HRV was related to reactive aggression, whereas the opposite physiological pattern (low HR, low stress SCL, high stress HRV) was related to proactive aggression. Furthermore, high stress SCL was related to anxiety symptoms, whereas low stress SCL was related to attention problems. These findings are important because they indicate heterogeneity within boys with ODD/CD and highlight the importance of using physiology to differentiate boys with different ODD/CD subtypes.

## Introduction

Aggression in young children is quite common [57]. When children grow older most of them learn to regulate this behavior. Children who persist in displaying aggressive and antisocial behaviors are at risk for a variety of negative outcomes: delinquency, unemployment, depression, anxiety and other psychiatric problems to name a few [9]. When aggressive and antisocial behavior develops into a pervasive pattern, affecting diverse domains of children’s functioning, this is referred to as oppositional defiant disorder (ODD) or conduct disorder (CD), belonging to the diagnostic class ‘disruptive, impulse-control, and conduct disorders’ of the Diagnostic and Statistical Manual of Mental Disorders (DSM-5) [2].

In the last decade, there is increasing evidence that biological processes play an important role in aggressive and antisocial behavior in children [36, 40, 60]. This is evident in abnormal functioning of the autonomic nervous system (ANS), one of the main human stress regulating systems. The ANS consists of two systems: the parasympathetic nervous system (PNS) and the sympathetic nervous system (SNS). The PNS is involved in most daily activities promoting calm, vegetative activities, whereas the SNS becomes active when a stressor is perceived [49]. In times of stress, a nearly complete withdrawal of the vagus nerve, the main nerve of the PNS, occurs [44]. Metabolic demands are suppressed, facilitating fight–flight reactions by accelerating heart rate (HR) and activating sweat glands, which increase skin conductance level (SCL). In times of rest, the vagus nerve decelerates HR, facilitating social engagement [43]. HR is an indicator of both the PNS and SNS, whereas SCL reflects only SNS. Activity of the vagus nerve can be measured by the heart rate variability (HRV), the fluctuation in intervals between heart beats. During stress, HRV is thought to drop as a consequence of vagal withdraw. High resting HRV enables an individual to select from a greater amount of actions to react to environmental demands if needed and is thought to be indicative of adequate self-regulation skills [45]. Malfunctioning of this ANS system might place children at risk for emotional dysregulation and thus aggression [5].

The best replicated ANS parameter in children with antisocial behavior is a low HR during rest as well as during stress [40]. SCL has been found to be lower in children with conduct problems, but not in aggressive children in general [36]. Meta-analyses confirm the low arousal theory [60] concluding that children with ODD/CD have a low basic arousal level (low HR and SCL) and, therefore, seek stimulating activities (sensation seeking theory [71]) and do not fear the negative consequences of their dangerous/aggressive actions (fearlessness theory [46]). Lower basal HRV has been found in children with conduct problems [6, 7, 38] as well as during (physical) stress in an aggressive community sample [11, 54].

Although there is clear evidence pointing towards reduced arousal and regulation in children with ODD/CD, some contradicting findings have been reported as well. Higher resting HR in children with ODD/CD [19, 70] as well as no difference in resting HR have been reported [18, 26]. SCL only seems to be lower in children diagnosed with CD [36]. Some studies found that baseline HRV was not associated with externalizing problem behavior or aggression [11, 54], whereas others found that externalizing problem behavior was associated with high baseline HRV [21]. Low HRV during stress was found to be associated with antisocial and aggressive behavior in a non-clinical sample [11, 54], but not in a clinical sample [6, 7]; one study found that high stress HRV was related to aggression in a non-clinical sample [21].

It is important to address these conflicting findings and examine the possibility that different arousal profiles may exist within children with ODD/CD, with some children showing over-arousal and some under-arousal. This is supported by observations that on a behavioral level there are also differences in type of behavioral/emotional problems, with some children, for example, behaving more reactively aggressive than others. Reactive or ‘hotblooded’ aggression is emotional and hostile, and elicited in response to perceived threat, provocation or frustration [22, 34]. Proactive or ‘coldblooded’ aggression on the other hand is goal directed or instrumental aggression. These subtypes of aggressive behaviors often correlate highly, but what is driving these behaviors is thought to be distinct [41]. This is, for example, expressed in differential deficits in underlying biology; proactive aggression is associated with reduced physiological arousal [34], while enhanced HR and SC response is associated with reactive aggression [56].

Furthermore, studies examining comorbidity suggest that boys with ODD/CD and comorbid ADHD have higher baseline HRs than boys with ADHD alone [62], but do not differ during stress or in SCL. Other studies found HR not to be different in those with CD or ODD and ADHD comorbidity [30, 59], nor did they differ in SCL [30]. Still others did find SCL to be attenuated in an ODD/ADHD sample [16]. Another study found that boys with CD and CD/ADHD had lower HR than controls, but boys with pure ADHD did not differ from boys with CD with or without comorbid ADHD or controls [29]. Baseline HRV was found to be lower in adolescent boys with CD/ADHD compared to controls, but not between boys with CD/ADHD and pure ADHD, or between boys with pure ADHD and controls [8]. Yet others did not find HRV to be different between an ODD/ADHD group and controls [16].

Children with ODD/CD can also suffer from comorbid anxiety. Higher resting HR and SCL have been found in a community sample displaying aggression and anxiety [38, 48] and during stress in anxious children without aggression [20, 68]. Internalizing problems have also been found to be associated with higher HR and lower HRV [21], and both anxiety and reactive aggression have been linked to autonomic over-arousal [10].

In addition, aggression is displayed in over fifty percent of children with autism spectrum disorder (ASD) [37], and one in four meet diagnostic criteria for either ODD or CD [33]. ASD has been associated with higher resting HRs than typically developing children [4] and lower HRV compared to controls [39, 63]. Studies on SCL in children with ASD reveal mixed results, with some finding low resting SCLs [58] and others high resting SCLs [31, 53]. Until now, studies focusing on ODD/CD have not studied ASD comorbidity in relation to ANS functioning.

The current study examined individual differences in ANS dysfunction in children with ODD/CD to help explain variability in behavioral phenotype within children with ODD/CD. Rather than focusing on aggression alone, this study also examines a range of emotional/behavioral problems (anxiety, attention and ASD traits). So far ASD traits have not been taken into account in studying boys with ODD/CD and arousal. We measured baseline arousal (HR, HRV, SCL) as well as arousal under stress. In line with earlier studies, we first compared the ODD/CD group with a control group. However, and crucial to our aims, our primary focus was on variability in the ODD/CD group. We think it is important to focus on heterogeneity in neurobiology, i.e., differences in arousal within ODD/CD, as this might help understand differential developmental risk in individual children. For example, some children exhibit heightened SNS activity (e.g., high SCL, low HRV) and may be especially vulnerable to stressful situations because their system is already ‘primed’ [28], causing greater risk for displaying reactive aggression [10]. Therefore, we examined whether individual differences in behavioral phenotype in the ODD/CD group (e.g., proactive/reactive aggression, anxiety, attention problems and ASD traits) were related to individual differences in functioning of the ANS system.

## Methods

The current study was approved by the Medical Ethical Committee of Leiden University Medical Centre (LUMC).

### Participants

#### DBD group

Inclusion criteria for the ODD/CD group were a diagnosis of ODD and/or CD on the Diagnostic Interview Schedule for Children (DISC-IV) [24], an estimated Intelligence Quotient (IQ) >70, and aged between 8 and 12 years; this resulted in a ODD/CD group of 66 boys. All boys met criteria for ODD diagnosis and 22 boys (33 %) also met CD criteria. Other comorbid diagnoses were: ADHD (*n* = 46, 68 %), anxiety (*n* = 39, 59 %), depression (*n* = 9, 14 %), ASD traits (mild: *n* = 22, 33 %, severe: *n* = 21, 32 %), and other disorders such as eating and tic disorders (*n* = 18, 27 %). Twenty-five boys (38 %) used psychostimulants and four (6 %) used atypical antipsychotics. For other demographic characteristics of the ODD/CD group see Table 1.

**Table 1:** Descriptive statistics for the ODD/CD (*n* = 66) and NC groups (*n* = 36)

	ODD/CD	NC	*t*	*p*
Age	10.3 ± 1.28	10.0 ± 1.25	1.13	.260
IQ	95.4 ± 14.39	103.8 ± 12.46	−2.97	.004
			*χ*	*p*
Native Dutch (%)	41 (62 %)	24 (67 %)	.21	.648

#### Non-clinical control group

Inclusion criteria for the non-clinical control (NC) group were estimated IQ >70, aged between 8 and 12 years, no medication use, and no aggression, expressed as a diagnosis of ODD or CD, a score outside the normal range (T > 60) on the externalizing scale of the Child Behavior Checklist (CBCL/6–18) or Teacher Report Form (TRF/6–18) [1]; this resulted in an NC group of 36 boys. Demographic characteristics of the NC group are displayed in Table 1.

### Recruitment and procedures

Boys with ODD/CD were recruited at clinical health centers (*n* = 22), special education schools (*n* = 32) and regular elementary schools (*n* = 12). NCs were recruited at regular elementary schools (*n* = 36). Boys referred through clinical centers were first screened with the CBCL [64]. Those who scored above the borderline cutoff point on the externalizing scale were administered the DISC-IV interview [24]. Participating boys were asked to visit Leiden University for 1 day with one of their parents. During this day parents signed an informed consent, filled out questionnaires and completed the DISC-IV interview. Boys completed computer tasks, physiological measures and filled out questionnaires. Within 2 weeks the second session took place either at the child’s school or at the clinical health center. The teacher of the child filled out the TRF [65] and the Instrument for Reactive and Proactive Aggression (IRPA) [42] questionnaire afterwards.

### Measures

Inclusion of the study was based on the Dutch version [24] of the DISC-IV interview [55] with one of the parents and the Dutch versions of the CBCL [64] and TRF [65] questionnaires [1]. The DISC is a highly structured diagnostic instrument [55] and was conducted by a clinical trained psychologist with experience. The subscales ‘rule breaking behavior’ and ‘aggressive behavior’ of the CBCL (parent report) and TRF (teacher report) were used to include controls that scored in the normal range (T < 60) on these subscales. Coefficient alpha’s (α) were .71 (CBCL) .79 (TRF) for ‘rule breaking behavior’ and .95 (CBCL) and .96 (TRF) for ‘aggressive behavior’.

IQ was measured with Vocabulary and Block Design, two subtests of the Dutch version [35] of the Wechsler Intelligence Scale for Children (WISC-IV) [67]. These subtests have been found to provide a good estimation of full-scale IQ scores [50].

Anxiety and attention problems were measured using the TRF [65]. Two subscales were used: anxious/depressed (α = .86) and attention problems (α = .95). Raw scores were transformed into T scores providing normal (<65), borderline (65–70) and clinical scores (70>).

ASD traits were measured with the Dutch version [47] of the Social Responsiveness Scale (SRS), a parent questionnaire for assessing autistic traits [14]. The SRS has good validity [13], and has good reliability [14]. Using the manual, total scores of severity of social deficits in autism spectrum conditions were transformed into T scores providing normal (<60), moderate (60–75) and clinical scores (75>). Coefficient alpha was .83.

Reactive and proactive aggression were measured by the IRPA [42]. Teachers reported about the frequency and the form of the aggressive behavior: proactive (3 items, e.g., intended to hurt or be mean to another child) or reactive (3 items, e.g., because someone teased or upset the child) on a five-point scale (never–always). The IRPA has good discriminant, convergent and construct validity [42]. Coefficient alpha was .87 for both reactive and proactive aggression.

Cardiac autonomic functioning (ECG) and SCL were assessed by a 24 bipolar channel Porti-system from TMSi (Oldenzaal, Netherlands) at a sample frequency of 512 Hz. For ECG measures a pre-high-pass filter of .5 Hz (ECG) and pre-low-pass filter of 3 Hz (SCL) were used. Before attaching the pre-gelled disposable ECG electrodes on the chest (sternum-V6 lead), the locations of the electrode placement were cleaned with alcohol. HR was measured in beats per minute. Heart rate variability (HRV) was measured using the square root of the mean squared differences (RMSSD) between adjacent N–N intervals. SCL was monitored using electrodes filled with electrode gel, taped to the medial phalanx surfaces of the middle and ring finger of the non-dominant hand. A high-pass filter of .07 Hz and a low-pass filter of .33 Hz were used to isolate SCLs (removing tonic changes, slow drifts and high-frequency noise). HR, HRV and SCL were calculated with Acqknowledge version 4.3.1.

Baseline and stress ECG and SCL were measured for 3 min whilst boys were sitting in a comfortable chair and watching a relaxing video. Stress was induced when boys were led to believe that they were competing against a videotaped opponent for the best performance and a highly favored award (for details, see [61]). Boys had to complete a simple computer task in which a random selection of 16 of the 55 trials was delayed by 6–12 s, causing frustration. They received negative feedback on their performance by the opponent. ECG and SCL were subsequently measured twice for 1 min at the start of two computer-based competitive decision making tasks involving reward and punishment, i.e., the ‘Door-opening task’ (for details, see [17]) and ‘Hungry Donkey task’ (for details, see [15]). Boys were led to believe they were playing against their opponent for best performance. After each task, the experimenters exchanged results, with the boys always losing both tasks. The ECG and SCL first minute registrations of both tasks were combined to create one ECG and SCL stress measure.

Stress manipulation was checked with a self-report scale containing twelve moods (happy, well, cheerful, good, liked, satisfied, afraid, worried, embarrassed, ashamed, angry, in control) which boys rated on a five-point scale ranging from positive towards negative feelings (e.g., 1 = happy, 5 = gloomy). This questionnaire is an adaptation of the Clinical self-rating scales of Von Zerssen [66]. All moods were combined into one negative mood score. Boys rated their moods twice, once after the baseline and once after the stress condition. Coefficient alphas were .77 for baseline and .90 for stress.

### Statistical analysis

First, data were checked with a missing data analysis. No patterns were revealed; therefore, all 66 boys with ODD/CD and 36 NC boys were included in the analysis. Then data were checked for normal distributions; for group comparisons (ODD/CD versus NC) the following variables were not normally distributed and successfully log transformed: HRV and SCL baseline and stress, reactive and proactive aggression and anxiety. *T* test revealed that boys with and without medication did not differ on any arousal or emotional/behavioral measures, and medication was, therefore, not controlled for in subsequent analyses. Proactive and reactive aggression correlated significantly with IQ. ANCOVAs, controlling for IQ, did not reveal different outcomes than *t* tests without controlling for IQ, therefore, *t* tests were performed to compare the ODD/CD group to NC group on aggression, anxiety, attention problems and ASD traits. Cohen’s *d* effect sizes were calculated with .2 being a small, .5 a medium and .8 a large effect [12]. Next, boys with ODD/CD were compared to NC boys on their baseline arousal level, followed by three independent repeated measures ANOVAs to test the effect of stress on HR, HRV and mean SCL as well as the effect of group (ODD/CD or NC) and the interaction effect of these two factors. Finally, a stepwise hierarchical regression analysis was carried out to explore the relationship of the arousal measures with anxiety, ASD traits, attention problems and aggression in boys with ODD/CD only. Anxiety was entered in the first step of the regression, followed by all other variables, because anxiety plays an important role in explaining arousal levels [21, 38, 48].

## Results

### Emotional/behavioral problems

The ODD/CD group and NC group were compared on symptoms of anxiety, attention problems, ASD traits, reactive and proactive aggression. One teacher did not return the IRPA (ODD/CD group) and 14 did not return the TRF questionnaire (8 in the ODD/CD group and 6 in the NC group). We were not able to collect the SRS of two boys in the ODD/CD group. The ODD/CD group scored significantly higher on both aggression measures as well as on all other measures than the NC group (see Table 2). All significant results were of large effect size.

**Table 2:** Means, SDs and *t* test statistics for ODD/CD and NC groups for aggression and emotional/behavioral problems

	ODD/CD	NC	*t*	*p*	Cohen’s *d*
	M ± SD	M ± SD			
Anxiety (TRF)	6.9 ± 4.79	2.5 ± 2.49	3.62	.001	1.15
Attention (TRF)	23.8 ± 11.62	7.2 ± 8.20	6.97	<.001	1.65
ASD traits (SRS)	69.9 ± 30.66	21.9 ± 12.28	8.99	<.001	2.06
Reactive aggression (IRPA)	21.3 ± 18.35	3.4 ± 6.18	6.69	<.001	1.31
Proactive aggression (IRPA)	13.6 ± 14.11	2.4 ± 4.11	5.46	<.001	1.08

*TRF* teacher report form, *SRS* Social Responsiveness Scale, *IRPA* Instrument for Reactive and Proactive Aggression

### Arousal

#### Baseline

First, the ODD/CD and NC group were compared on the three baseline measures of arousal with a *t* test. Baseline measures were incomplete for two boys with ODD/CD and one control. The ODD/CD group had a higher baseline HR than the NC group, *t* = 2.30, *p* = .024, with a medium effect, *d* = .49, but HRV and SCL did not differ between both groups (see Table 3).

**Table 3:** Means, SDs in the ODD/CD and NC groups for mood and arousal levels during baseline and stress

	Baseline		Stress		*p*	*η* ^*2*^
	M ± SD		M ± SD			
	ODD/CD	NC	ODD/CD	NC		
Mood	3.3 ± .59	3.1 ± .58	2.5 ± 1.06	2.6 ± .89	<.001	.26
HR	75.3 ± 10.31	70.6 ± 8.68	94.3 ± 14.53	90.8 ± 12.75	<.001	.77
HRV	71.0 ± 36.82	77.9 ± 38.24	39.0 ± 22.73	47.3 ± 27.33	<.001	.60
SCL	6.4 ± 4.78	6.1 ± 2.87	10.5 ± 8.40	10.1 ± 5.79	<.001	.48

*HR* heart rate expressed as beats per minute, *HRV* heart rate variability expressed as milliseconds (ms), *SCL* skin conductance level expressed as micro Siemens (μS)

#### Stress

Next, stress manipulation was checked by analyzing mood change from baseline to stress condition. There was a significant main effect of stress, *F*(1, 96) = 33.69, *p* < .001, with mood significantly decreasing from baseline to stress (see Table 3). There were no group or stress by group interaction effects (*p* > .05).

With respect to stress, four boys of the ODD/CD group did not complete all stress HR and HRV measures and five did not complete SCL measurement. In the NC group three SCL measures were incomplete. For all three arousal measures there was a significant main effect of stress, showing that the stress manipulation was effective. HR (*F*(1, 97) = 316.30, *p* < .001) and SCL (*F*(1, 88) = 40.45, *p* < .001) significantly increased, whereas HRV (*F*(1, 94) = 79.45 *p* < .001) significantly decreased from baseline to stress (see Table 3). There were no group or stress by group interaction effects for any of the dependent measures (*p* > .05).

### Arousal and emotional/behavioral problems within the DBD group

The correlation matrix showed that anxiety correlated positively with attention and reactive aggression (see Table 4). Proactive aggression showed a positive correlation with attention and a negative correlation with ASD traits. Furthermore, reactive and proactive aggression correlated positively with each other.

**Table 4:** Correlation matrix of anxiety, attention, ASD traits and aggression (*r*)

	Anxiety	Attention	ASD traits	Reactive aggression
Anxiety				
Attention	.42**			
ASD traits	.08	−.06		
Reactive aggression	.32*	.22	−.06	
Proactive aggression	.25	.38**	−.29*	.44**

* Correlation is significant at the .05 level (two tailed)

** Correlation is significant at the .01 level (two tailed)

Finally, a stepwise linear regression analysis was performed to explore the relationship of arousal and variability of emotional/behavioral problems within boys with ODD/CD.

#### Baseline

The results indicated that Model 1, with anxiety as the only predictor, was not significant for HR (*p* = .567), HRV (*p* = .305) or SCL (*p* = .068). Model 2 was significant for HR [*R*^*2*^ = .19, *F*(5, 50) = 2.38, *p* = .051] and SCL [*R*^*2*^ = .20, *F*(5, 50) = 2.42, *p* = .048], but not for HRV (*p* = .228). Significant predictors for HR were: proactive (*β* = −.38, *p* = .018) and reactive aggression (*β* = .35, *p* = .022). For SCL the significant predictor was reactive aggression (*β* = .34, *p* = .023). All other predictors were not significant (see Table 5). In other words, low resting HR was associated with proactive aggression, whereas high resting HR and SCL was associated with reactive aggression.

**Table 5:** Regression coefficients (*β*) for all three arousal measures during baseline and stress within the ODD/CD group

	Baseline			Stress		
	HR	HRV	SCL	HR	HRV	SCL
Step 1						
Anxiety	.08	.14	.25	−.00	.22	.16
Step 2						
Anxiety	.11	.01	.22	.01	.18	.32*
Attention	−.12	.27	−.17	.05	.07	−.32*
ASD traits	.02	−.03	.13	−.01	.04	−.01
Reactive aggression	.35*	−.06	.34*	.29	−.30	.19
Proactive aggression	−.38*	.17	−.10	−.48**	.41**	−.34*

*HR* heart rate expressed as beats per minute, *HRV* heart rate variability expressed as milliseconds (ms), *SCL* skin conductance level expressed as micro Siemens (μS)

* Correlation is significant at the .05 level (two tailed)

** Correlation is significant at the .01 level (two tailed)

#### Stress

The results indicated that Model 1 was not significant for HR (*p* = .984), HRV (*p* = .108), and SCL (*p* = .268). Model 2 was significant for HRV [*R*^*2*^ = .21, *F*(5, 48) = 2.54, *p* = .041], and SCL [*R*^*2*^ = .25, *F*(5, 44) = 2.99, *p* = .021]. Significant predictors for HRV were proactive (*β* = .41, *p* = .012) and reactive aggression (β = −.30, *p* = .051). For SCL the significant predictors were anxiety (*β* = .32, *p* = .041), attention problems (*β* = −.32, *p* = .039) and proactive aggression (*β* = −.34, *p* = .040). All other predictors were not significant (see Table 5). Although proactive aggression (*β* = −.48, *p* = .004) was a significant predictor for stress HR in step 2, the model was not significant [*R*^*2*^ = .18, *F*(5, 49) = 2.14, *p* = .077]. High stress HRV was associated with more proactive aggression and low stress HRV was associated with more reactive aggression. High stress SCL was associated with more anxiety, and low stress SCL was associated with more attention problems and proactive aggression.

## Discussion

It is often reported that children with oppositional defiant disorder (ODD) or conduct disorder (CD) are under-aroused. However, the evidence is mixed with some children with ODD/CD displaying high arousal. This has led to the hypothesis that various profiles of ANS dysfunction may exist within children with ODD/CD. This knowledge is important to explain individual differences in behavioral phenotype in boys with ODD/CD. Our groupwise comparisons of boys with ODD/CD and controls revealed that boys with ODD/CD had a higher baseline HR, but did not differ in stress HR, HRV or SCL. However, focusing on the individual differences within boys with ODD/CD revealed that high arousal (high baseline HR and SCL and, low stress HRV) was related to more problems in reactive aggression, whereas low arousal (low HR, low stress SCL and, high stress HRV) was associated with more problems in proactive aggression. Furthermore, high stress SCL was associated with more anxiety problems, whereas low stress SCL was related to more attention problems.

First, we focused on the group differences between boys with ODD/CD and controls in aggression, anxiety, attention problems and ASD traits. Boys with ODD/CD had higher levels on all measures. The DISC interview [24] also revealed that within the ODD/CD group comorbid disorders were present: 70 % ADHD, 59 % anxiety, 14 % depression, and 27 % other disorders such as eating and tic disorders. Interestingly, groupwise comparisons on the physiology measures showed that the ODD/CD group had a higher baseline HR than controls. High baseline HR has also been reported by De Wied, et al. [19] and Zahn and Kruesi [70]. This is in contrast to the finding that children with antisocial behavior have a low HR [40]. The higher baseline HR of the ODD/CD group could have been caused by the higher levels of anxiety in this group; however, regressions showed that this was not the case. Increased levels of HR could not be explained by high anxiety in the current study.

We did not find differences in baseline HRV between boys with ODD/CD and controls. Our results are unexpected, but similar to Calkins, et al. [11] and Scott and Weems [54]. Others did find baseline HRV to be different between boys with ODD/CD and controls [6, 7, 21, 38]. During stress we did not find group differences either, in line with Beauchaine et al. [6], and Beauchaine et al. [7], but contrary to Calkins et al. [11], Dietrich et al. [21] and Scott and Weems [54]. These conflicting results in HRV across studies may be explained by the studies different stress conditions. Some used supine (rest) versus standing position (stress) [21, 38], whereas others used watching a relaxing video clip (rest) versus mental arithmetic task (stress) [54] or watching an emotional video of an argument (stress) [6]. Dietrich et al. [21] further suggest that this difference in findings might be caused by a community sample versus a high risk sample (clinical). Another possibility is that HRV may only relate to certain types of aggression. Low HRV is associated with emotion dysregulation and may, therefore, relate to reactive aggression, the emotional and hostile form of aggression [52]. The above-mentioned studies have not taken different types of aggression into account and focused on group differences only. Therefore, our other aim of this study was to examine whether ANS functioning was related to type of aggression and specific behavioral/emotional problems within the ODD/CD group.

Thus, second we focused on individual differences within boys with ODD/CD rather than the ODD/CD group as a whole. These analyses showed specific arousal profiles: high arousal (high baseline HR and SCL and, low stress HRV) was related to more problems in reactive aggression, whereas low arousal (low HR, low stress SCL and, high stress HRV) was associated with more problems in proactive aggression. The association between high arousal and reactive aggression is in line with the anger–frustration theory of reactive aggression: reactive aggression is related to sympathetic over-arousal in response to perceived threat or provocation [69]. The association between low HR and high proactive aggression is consistent with the under-arousal model of proactive aggression: the state of low arousal might feel unpleasant and, therefore, one seeks stimulating activities such as proactive aggression. Xu et al. [69] also reported a relation between low baseline HR and high proactive aggression, but they found this relationship for reactive aggression too. Yet another study did not find such a relationship between baseline HR and reactive or proactive aggression [51]. The anger–frustration theory of reactive aggression specifically refers to situations eliciting anger, though one can argue that if the system is already primed to under regulate emotions and behavior (at rest) it would get only worse adding stress. This was indeed found, during baseline HRV was not related to aggression, but during stress it was: low HRV (e.g., low regulation) was associated with more reactive aggression, while high HRV was related to high proactive aggression. Although proactive aggression is thus associated with increased regulation and this is typically considered adaptive, in this case it may be used for antisocial activities. Two recent studies focusing on community samples, rather than clinical samples, also linked specific HRV patterns to subtypes of aggression; in contrast to our baseline HRV finding, Scarpa et al. [51] found that low baseline HRV was associated with reactive aggression, whereas high baseline HRV was associated with proactive aggression in a mixed gender sample. Xu et al. [69] also found that low baseline HRV was associated with high levels of reactive aggression, but they did not find proactive aggression to be related to HRV. We did not find a relation between HRV and reactive or proactive during baseline, but we did during stress in a ODD/CD sample. In line with Hubbard et al. [32], during stress SCL was associated with proactive aggression. In contrast to Hubbard, et al. [32] we did not find reactive aggression to be related to high SCL during stress, but we did find this relationship during baseline. Although Hubbard et al. [32] did not report baseline measures of SCL, Scarpa et al. [51] did, yet found the opposite pattern in comparison to our study; high SCL was associated with proactive aggression and low SCL with reactive aggression. Recently, these differential patterns of SCL in relation to type of aggression were also illustrated in a study showing that proactive aggression, but not reactive aggression, was related to abnormal reactions in SCL during a fear condition task [25].

Furthermore, high stress SCL was associated with more anxiety problems, whereas low stress SCL was related to more attention problems. Previous studies already reported this association of high arousal and anxiety [38, 48]. These results may help us explain comorbidity in childhood disorders; for example, the high rate of comorbid anxiety disorders within ODD/CD. Scarpa et al. [51] suggested that low HRV indicates a dysregulated affective style and emotional inflexibility, which disorders such as depression, anxiety and aggression have in common. The finding that low SCL and high levels of attention problems were associated in the current study have been reported before [16], demonstrating that attention problems share an autonomic profile with proactive aggression, though others did not find SCL to be related to ADHD [29, 30, 62].

Because previous research has found that children with ASD have attenuated HRVs [39, 63] and over fifty percent exhibit aggressive behavior [37], we expected that high levels of arousal might be explained by increased levels of ASD traits within ODD/CD. Although ASD traits were increased in this group (34 % had mild and 34 % had severe ASD traits), ASD traits were not related to any of the physiological measures. Thus high arousal is typical for some children with ODD/CD and is not explained by comorbid ASD traits. Instead symptoms of anxiety and attention problems were related to specific and different physiological profiles.

This study has some limitations that need to be acknowledged. The ODD/CD group was a diverse one in that they scored not only high on aggression but also on other emotional/behavioral problems. This may have caused some contradicting findings with previous literature that have included children with ODD/CD without comorbidity. We think that this variation in behavioral phenotype may help us understand the complex relationship between neurobiology and behavior underlying psychopathology and chose to include all boys with ODD/CD regardless of any comorbid disorders. Another issue is that parents and their sons had to visit Leiden University for one day. This may have biased our sample in that only highly motivated parents took part. However, we did not rely on parent questionnaires only; we also used teachers as informants. Finally we included only boys in our sample. Problems with aggressive and antisocial behavior are not unique to boys, they have been found in girls as well (e.g., [7]). It would be interesting to study the relationship of ANS functioning and emotional/behavioral problems in girls in future studies.

Taken together, physiology is differentially related to the type of aggression within ODD/CD as well as to other specific emotional/behavioral problems they experience. These insights are important because it illustrates diversity within boys with ODD/CD and issues the need to match the individual profile of the child to treatment, and thus contribute to more effective treatment results. Two recent studies found that treatment outcome can be predicted by physiological measures. Gatzke-Kopp et al. [27] found in a preschool community sample that low HRV reactivity to anger was associated with more externalizing problem behavior and less emotion-regulation skills, whereas low HRV reactivity to fear was associated with more improvement after an intervention targeting social skills. Bagner et al. [3] found in a sample of three-year-olds that low baseline HRV was associated with less disruptive behavior after treatment, indicating that those with less regulation skills have more to gain from treatment. These studies show how physiological assessment can help us in predicting treatment outcome and thus improve treatment effectiveness. This is a promising field for future research, especially given that there is very little evidence-based understanding of how and why treatments produce change [23], so there is still much to gain.

## References

[1] Achenbach TM, Rescorla LA (2001) Manual for the ASEBA school-age forms and profiles. In: University of Vermont, Research Center for Children, Youth, and Families, Burlington

[2] American Psychiatric Association (2013) The Diagnostic and Statistical Manual of Mental Disorders (5th ed.). American Psychiatric Publishing, Arlington

[3] Bagner DM, Graziano PA, Jaccard J, Sheinkopf SJ, Vohr BR, Lester BM (2012). An initial investigation of baseline respiratory sinus arrhythmia as a moderator of treatment outcome for young children born premature with externalizing behavior problems. Behav Ther.

[4] Bal E, Harden E, Lamb D, Van Hecke AV, Denver JW, Porges SW (2010). Emotion recognition in children with autism spectrum disorders: relations to eye gaze and autonomic state. J Autism Dev Disord.

[5] Beauchaine T (2001). Vagal tone, development, and Gray’s motivational theory: toward an integrated model of autonomic nervous system functioning in psychopathology. Dev Psychopathol.

[6] Beauchaine TP, Gatzke-Kopp L, Mead HK (2007). Polyvagal Theory and developmental psychopathology: emotion dysregulation and conduct problems from preschool to adolescence. Biol Psychol.

[7] Beauchaine TP, Hong J, Marsh P (2008). Sex differences in autonomic correlates of conduct problems and aggression. J Am Acad Child Adolesc Psychiatry.

[8] Beauchaine TP, Katkin ES, Strassberg Z, Snarr J (2001). Disinhibitory psychopathology in male adolescents: discriminating conduct disorder from attention-deficit/hyperactivity disorder through concurrent assessment of multiple autonomic states. J Abnorm Psychol.

[9] Bradshaw CP, Schaeffer CM, Petras H, Ialongo N (2010). Predicting negative life outcomes from early aggressive-disruptive behavior trajectories: gender differences in maladaptation across life domains. J Youth Adolesc.

[10] Bubier JL, Drabick DA (2009). Co-occurring anxiety and disruptive behavior disorders: the roles of anxious symptoms, reactive aggression, and shared risk processes. Clin Psychol Rev.

[11] Calkins SD, Graziano PA, Keane SP (2007). Cardiac vagal regulation differentiates among children at risk for behavior problems. Biol Psychol.

[12] Cohen J (2013). Statistical power analysis for the behavioral sciences.

[13] Constantino JN, Davis SA, Todd RD, Schindler MK, Gross MM, Brophy SL, Metzger LM, Shoushtari CS, Splinter R, Reich W (2003). Validation of a brief quantitative measure of autistic traits: comparison of the social responsiveness scale with the autism diagnostic interview-revised. J Autism Dev Disord.

[14] Constantino JN, Gruber CP (2005). Social responsiveness scale (SRS) manual.

[15] Crone E, van der Molen M (2004). Developmental changes in real life decision making: performance on a gambling task previously shown to depend on the ventromedial prefrontal cortex. Dev Neuropsychol.

[16] Crowell SE, Beauchaine TP, Gatzke-Kopp L, Sylvers P, Mead H, Chipman-Chacon J (2006). Autonomic correlates of attention-deficit/hyperactivity disorder and oppositional defiant disorder in preschool children. J Abnorm Psychol.

[17] Daugherty T, Quay H (1991). Response perseveration and delayed responding in childhood behavior disorders. J Child Psychol Psychiatry.

[18] De Wied M, Van Boxtel AV, Matthys W, Meeus W (2012). Verbal, facial and autonomic responses to empathy-eliciting film clips by disruptive male adolescents with high versus low callous-unemotional traits. J Abnorm Child Psychol.

[19] De Wied M, Van Boxtel AV, Posthumus JA, Goudena PP, Matthys W (2009). Facial EMG and heart rate responses to emotion-inducing film clips in boys with disruptive behavior disorders. Psychophysiology.

[20] Dieleman GC, Huizink AC, Tulen JH, Utens EM, Creemers HE, Van Der Ende J, Verhulst FC (2014). Alterations in HPA-axis and autonomic nervous system functioning in childhood anxiety disorders point to a chronic stress hypothesis. Psychoneuroendocrinology.

[21] Dietrich A, Riese H, Sondeijker FE, Greaves-Lord K, Van Roon AM, Ormel J, Neeleman J, Rosmalen JG (2007). Externalizing and internalizing problems in relation to autonomic function: a population-based study in preadolescents. J Am Acad Child Adolesc Psychiatry.

[22] Dodge KA, Pepler DJ, Rubin KH (1991). The structure and function of reactive and proactive aggression. The development and treatment of childhood aggression.

[23] Eyberg S, Nelson M, Boggs S (2008). Evidence-based psychosocial treatments for children and adolescents with disruptive behavior. J Clin Child Adolesc Psychol.

[24] Ferdinand RF, van der Ende J (2002). Nederlandse vertaling van de disc-iv. Diagnostic Interview Schedule for Children.

[25] Gao Y, Tuvblad C, Schell A, Baker L, Raine A (2015). Skin conductance fear conditioning impairments and aggression: a longitudinal study. Psychophysiology.

[26] Garralda ME, Connell J, Taylor DC (1991). Psychophysiological anomalies in children with emotional and conduct disorders. Psychol Med.

[27] Gatzke-Kopp LM, Greenberg M, Bierman K (2013). Children’s parasympathetic reactivity to specific emotions moderates response to intervention for early-onset aggression. J Clin Child Adolesc Psychol.

[28] Gatzke-Kopp LM, Greenberg MT, Fortunato CK, Coccia MA (2012). Aggression as an equifinal outcome of distinct neurocognitive and neuroaffective processes. Dev Psychopathol.

[29] Herpertz SC, Mueller B, Wenning B, Qunaibi M, Lichterfeld C, Herpertz-Dahlmann B (2003). Autonomic responses in boys with externalizing disorders. J Neural Transm.

[30] Herpertz SC, Wenning B, Mueller B, Qunaibi M, Sass H, Herpertz-Dahlmann B (2001). Psychophysiological responses in ADHD boys with and without conduct disorder: implications for adult antisocial behavior. J Am Acad Child Adolesc Psychiatry.

[31] Hirstein W, Iversen P, Ramachandran VS (2001). Autonomic responses of autistic children to people and objects. Proc Roy Soc Biol Sci.

[32] Hubbard JA, McAuliffe MD, Morrow LJ (2010). Reactive and proactive aggression in childhood and adolescence: precursors, outcomes, processes, experiences, and measurement. J Pers.

[33] Kaat AJ, Lecavalier L (2013). Disruptive behavior disorders in children and adolescents with autism spectrum disorders: a review of the prevalence, presentation, and treatment. Res Autism Spectrum Disord.

[34] Kempes M, Matthys W, de Vries H, van Engeland H (2005). Reactive and proactive aggression in children–a review of theory, findings and the relevance for child and adolescent psychiatry. Eur Child Adolesc Psychiatry.

[35] Kort W, Schittekatte M, Dekker PH, Verhaeghe P, Compaan EL, Bosmans M, Vermeir G (2005) WISC-III NL wechsler intelligence scale for children. Derde Editie NL. Handleiding en Verantwoording. Harcourt Test Publishers/Nederlands Instituut voor Psychologen, Amsterdam

[36] Lorber MF (2004). Psychophysiology of aggression, psychopathy, and conduct problems: a meta-analysis. Psychol Bull.

[37] Mazurek MO, Kanne SM, Wodka EL (2013). Physical aggression in children and adolescents with autism spectrum disorders. Res Autism Spectrum Disord.

[38] Mezzacappa E, Tremblay RE, Kindlon D, Saul JP, Arseneault L, Seguin J, Pihl RO, Earls F (1997). Anxiety, antisocial behavior, and heart rate regulation in adolescent males. J Child Psychol Psychiatry.

[39] Ming X, Julu PO, Brimacombe M, Connor S, Daniels ML (2005). Reduced cardiac parasympathetic activity in children with autism. Brain Dev.

[40] Ortiz J, Raine A (2004). Heart rate level and antisocial behavior in children and adolescents: a meta-analysis. J Am Acad Child Adolesc Psychiatry.

[41] Polman H, Orobio de Castro B, Koops W, van Boxtel HW, Merk WW (2007). A meta-analysis of the distinction between reactive and proactive aggression in children and adolescents. J Abnorm Child Psychol.

[42] Polman H, Orobio de Castro B, Thomaes S, van Aken M (2009). New directions in measuring reactive and proactive aggression: validation of a teacher questionnaire. J Abnorm Child Psychol.

[43] Porges SW (2007). The polyvagal perspective. Biol Psychol.

[44] Porges SW (2001). The polyvagal theory: phylogenetic substrates of a social nervous system. Int J Psychophysiol.

[45] Porges SW (1992). Vagal tone: a physiologic marker of stress vulnerability. Pediatrics.

[46] Raine A (1993). The psychopathology of crime: criminal behavior as a clinical disorder.

[47] Roeyers H, Thys M, Druart C, De Schryver M, Schittekatte M (2011). Screeningslijst voor autismespecturmstoornissen.

[48] Rogeness GA, Cepeda C, Macedo CA, Fischer C, Harris WR (1990). Differences in heart rate and blood pressure in children with conduct disorder, major depression, and separation anxiety. Psychiatric Res.

[49] Sapolsky RM (1998). Why zebras don’t get ulcers. The acclaimed guide to stress, stress-related diseases, and coping.

[50] Sattler JM (1992) Assessment of children (3rd edn, rev. and updated). J. M. Sattler., San Diego

[51] Scarpa A, Haden SC, Tanaka A (2010). Being hot-tempered: autonomic, emotional, and behavioral distinctions between childhood reactive and proactive aggression. Biol Psychol.

[52] Scarpa A, Tanaka A, Haden SC (2008). Biosocial bases of reactive and proactive aggression: the roles of community violence exposure and heart rate. J Commun Psychol.

[53] Schoen SA, Miller LJ, Brett-Green BA, Nielsen DM (2009). Physiological and behavioral differences in sensory processing: a comparison of children with autism spectrum disorder and sensory modulation disorder. Res Autism Spectrum Disord.

[54] Scott BG, Weems CF (2014). Resting vagal tone and vagal response to stress: associations with anxiety, aggression, and perceived anxiety control among youths. Psychophysiology.

[55] Shaffer D, Fisher P, Lucas CP, Dulcan MK, Schwab-Stone ME (2000). NIMH Diagnostic Interview Schedule for Children Version IV (NIMH DISC-IV): description, differences from previous versions, and reliability of some common diagnoses. J Am Acad Child Adolesc Psychiatry.

[56] Stadler C, Poustka F, Sterzer P (2010). The heterogeneity of disruptive behavior disorders: implications for neurobiological research and treatment. Front Psychiatry.

[57] Tremblay RE, Nagin DS, Seguin JR, Zoccolillo M, Zelazo PD, Boivin M, Perusse D, Japel C (2004). Physical aggression during early childhood: trajectories and predictors. Pediatrics.

[58] Van Engeland H, Roelofs JW, Verbaten MN, Slangen JL (1991). Abnormal electrodermal reactivity to novel visual stimuli in autistic children. Psychiatry Res.

[59] van Goozen SH, Matthys W, Cohen-Kettenis PT, Gispen-de Wied C, Wiegant VM, van Engeland H (1998). Salivary cortisol and cardiovascular activity during stress in oppositional-defiant disorder boys and normal controls. Biol Psychiatry.

[60] Van Goozen SHM, Fairchild G, Snoek H, Harold GT (2007). The evidence for a neurobiological model of childhood antisocial behavior. Psychol Bull.

[61] Van Goozen SHM, Matthys W, Cohen Kettenis P, Buitelaar JK, Van Engeland H (2000). Hypothalamic-pituitary-adrenal axis and autonomic nervous system activity in disruptive children and matched controls. J Am Acad Child Adolesc Psychiatry.

[62] Van Lang ND, Tulen JH, Kallen VL, Rosbergen B, Dieleman G, Ferdinand RF (2007). Autonomic reactivity in clinically referred children attention-deficit/hyperactivity disorder versus anxiety disorder. Eur Child Adolesc Psychiatry.

[63] Vaughan Van Hecke A, Lebow J, Bal E, Lamb D, Harden E, Kramer A, Denver J, Bazhenova O, Porges SW (2009). Electroencephalogram and heart rate regulation to familiar and unfamiliar people in children with autism spectrum disorders. Child Dev.

[64] Verhulst FC, Ende JD, Koot HM (1996) Handleiding voor de CBCL/4-18. Sophia Kinderziekenhuis, Erasmus MC., Rotterdam

[65] Verhulst FC, Ende Jvd, Koot HM (1997) Handleiding voor de Teacher’s Report Form (TRF). Sophia Kinderziekenhuis, Erasmus MC., Rotterdam

[66] Von Zerssen D (1986) Clinical self-rating scales (CSRS) of the Munich psychiatric information system (PSYCHIS München). In: Assessment of depression. Springer, p 270–303

[67] Wechsler D (2003). Wechsler Intelligence Scale for Children—4th Edition (WISC-IV^®^).

[68] Weems CF, Zakem AH, Costa NM, Cannon MF, Watts SE (2005). Physiological response and childhood anxiety: association with symptoms of anxiety disorders and cognitive bias. J Clin Child Adolesc Psychol.

[69] Xu Y, Raine A, Yu L, Krieg A (2014). Resting heart rate, vagal tone, and reactive and proactive aggression in Chinese children. J Abnorm Child Psychol.

[70] Zahn TP, Kruesi MJ (1993). Autonomic activity in boys with disruptive behavior disorders. Psychophysiology.

[71] Zuckerman M (1979). Sensation seeking: beyond the optimum level of arousal.

